# Elastic modulus data for additively and conventionally manufactured variants of Ti-6Al-4V, IN718 and AISI 316 L

**DOI:** 10.1038/s41597-023-02387-6

**Published:** 2023-07-20

**Authors:** Birgit Rehmer, Faruk Bayram, Luis Alexander Ávila Calderón, Gunther Mohr, Birgit Skrotzki

**Affiliations:** Bundesanstalt für Materialforschung und -prüfung (BAM), Berlin, Germany

**Keywords:** Metals and alloys, Mechanical properties

## Abstract

This article reports temperature-dependent elastic properties (Young’s modulus, shear modulus) of three alloys measured by the dynamic resonance method. The alloys Ti-6Al-4V, Inconel IN718, and AISI 316 L were each investigated in a variant produced by an additive manufacturing processing route and by a conventional manufacturing processing route. The datasets include information on processing routes and parameters, heat treatments, grain size, specimen dimensions, and weight, as well as Young’s and shear modulus along with their measurement uncertainty. The process routes and methods are described in detail. The datasets were generated in an accredited testing lab, audited as BAM reference data, and are hosted in the open data repository Zenodo. Possible data usages include the verification of the correctness of the test setup via Young’s modulus comparison in low-cycle fatigue (LCF) or thermo-mechanical fatigue (TMF) testing campaigns, the design auf VHCF specimens and the use as input data for simulation purposes.

## Background & Summary

In recent years, additive manufacturing (AM), also known as 3D printing, has made its way into production technology^1,2^. AM technologies are capable to produce final components in manufacturing processes where material cohesion is created layer-by-layer. This concerns not only metals, but also ceramics, glasses and polymers, and even composite materials^1–6^. AM processes offer high degrees of freedom for the design of (individual) components with a comparably short process chain. They are also attracting increasing interest as a potential repair process. Besides, AM works close to the final contour (net-shaped), so no or only little reworking is required. Several major R&D activities have been recognized in research institutes and industries for the last two decades.

Various AM metal processes are available^1,2^. Examples of metal AM processes are the laser-based powder bed fusion process PBF-LB/M and the laser-based direct energy deposition with powder as feedstock (DED-LB/M). (Note: PBF-LB/M and DED-LB/M are the abbreviations following the nomenclature defined in DIN EN ISO/ASTM 52900^7^ and replace the abbreviations L-PBF and DED-L.) Commonly processed metallic materials are stainless steel (AISI 316 L), titanium alloys (Ti-6Al-4V), and Ni-based superalloys (IN 718) for potential applications in aerospace, energy, medical, automotive, or at high temperatures^1^. The processes of the two outlined AM technologies are now well advanced and with the optimum manufacturing parameters matched to the material to be processed, it is possible to produce components with almost 100% density. Some important manufacturing parameters are the laser power and the scanning velocity, the beam diameter, the scan strategy (i.e., the strategy with which the beam track is guided over the surface, e. g., alternately lengthwise and crosswise), the applied powder layer thickness, and the quality of the powder itself. The substrate and its temperature on which the part is built also play a role. All these parameters (and some more) influence the density and occurrence of possible metallurgical defects, the final product’s microstructure, texture, and residual stresses.

AM processes of metals and alloys usually produce completely different microstructures than conventional manufacturing processes (e. g. rolling, extrusion). Grains or regions of them are often elongated in the building direction and the grain growth follows the local temperature gradient, which may result in anisotropic properties. In addition, the microstructure can span multiple length scales (from nm to sub-mm) as Wang *et al*.^8^ have shown for PBF-LB/M processed austenitic 316 L stainless steels. A high fraction of low-angle grain boundaries was observed. Further features include fusion boundaries, dendritic and cellular walls, dislocations, nm-size precipitates at cell walls, segregation of elements to the solidification cellular walls, and atomic scale impurities^8^. The multiscale microstructure causes locally different deformation behavior, which is often referred to as hetero deformation^9–11^.

It is well known that the columnar grain structure in AM materials may result in anisotropic mechanical properties, including elastic moduli. This phenomenon is also observed after other manufacturing routes, e.g. in directionally solidified or single crystal Ni-based superalloys, which are often used in applications (such as turbine blades) with <001> texture^12^. Single crystals of *pure nickel* are elastically anisotropic, therefore the Young’s modulus depends on crystal direction (all values for room temperature): E_<001>_ = 125 GPa, E_<110>_ = 220 GPa and E_<111>_ = 294 GPa^12^ (note that in fcc materials the modulus is usually lowest in <001> and highest in <111> direction). The Young’s modulus of *polycrystalline nickel* is given as 207 GPa^12^. Data for single crystalline *Ni-based superalloys* show a similar degree of anisotropy and are summarized e.g. in^13^. In AM Ni-based superalloys, the preferred grain growth direction follows the [001] direction parallel to the thermal gradient^14^. Usually, this preferred growth direction and the use of a given scan and deposition strategies results in a specific texture^15–17^. Young’s moduli in AM IN718 therefore often show different values in built direction and normal to it^18–20^. Directional Young’s modulus values for conventional IN718 are given by Kumara *et al*.^19^. For stainless austenitic steel, calculations of directional Young’s moduli based on elastic constants published by Ledbetter^21^ indicate even a higher anisotropy than for Ni-based alloys: E_<001>_ = 93.8 GPa, E_<110>_ = 193.5 GPa and E_<111>_ = 299.8 GPa. The modulus of AISI 316 L at room temperature for the polycrystalline material is given as 200 GPa (https://www.preschstahl.de/files/wd/1.4404.pdf). Single crystals of pure hcp Titanium also show high anisotropy: E_<0001>_ = 93.8 GPa and E_<11-20>_ = 145 GPa^22^. The Young’s modulus at room temperature is 110 GPa.

AM has reached a stage where first components are being transferred to service. Reliable material parameters such as strength parameters and Young’s modulus are required for future and ongoing component design. The Young’s modulus is a fundamental material parameter for material selection and design, and it is also needed for microstructure simulation (e. g. in crystal plasticity models). However, complete datasets that include the required process and microstructure information are still scarce. Young’s modulus is often derived (as a by-product) from tensile tests, but this often provides erroneous characteristic values^23^ due to poor alignment of the load train in a tensile testing machine and the use of one-sided strain measurement. Therefore, in the relevant standards, this value is not designated as “Young’s modulus” but as “straight line portion of the stress-strain curve”^24^, with the strain being determined by extensometer measurement^25^. Therefore, it would be valuable to have a high-precision Young’s and shear modulus dataset.

Young’s modulus can also be determined using dynamic methods, e.g., the resonance method, ultrasonic pulse technique or impulse excitation (cf. section 7.1.4 in Wiederhorn *et al*.^26^). The resonance method is based on the measurement of the resonance frequencies of a freely suspended specimen after excitation, e. g., by a piezo actuator. The method is very well suited for homogeneous materials that show elastic behavior. It is just as suitable for metallic materials as for advanced ceramics or glass and is covered by international standards, for example ASTM E1875 and DIN EN 843-2^27,28^. It allows the temperature-dependent determination of Young’s modulus and shear modulus with high accuracy and with only one specimen. The elastic modulus is calculated from the specimen geometry, the mass, and the measured resonant frequencies.

Considering the scarce availability of complete datasets for AM materials to date, this contribution presents values for the dynamic Young’s modulus and shear modulus for three different additively manufactured alloys and their conventional variants, which have been generated in an accredited testing lab, audited as BAM reference data and published in a data repository. The PBF-LB/M process for two materials (IN718 and 3176 L) was performed on the same machine in the same laboratory. The resonance measurements were performed on the same calibrated machine in the same laboratory according to the respective standard and using the same analysis procedure. Further mechanical properties as well as information on microstructure and residual stresses have been published elsewhere^20,29–33^ and can be combined with the elasticity values presented here.

## Methods

### Material

**Fig. 1:**
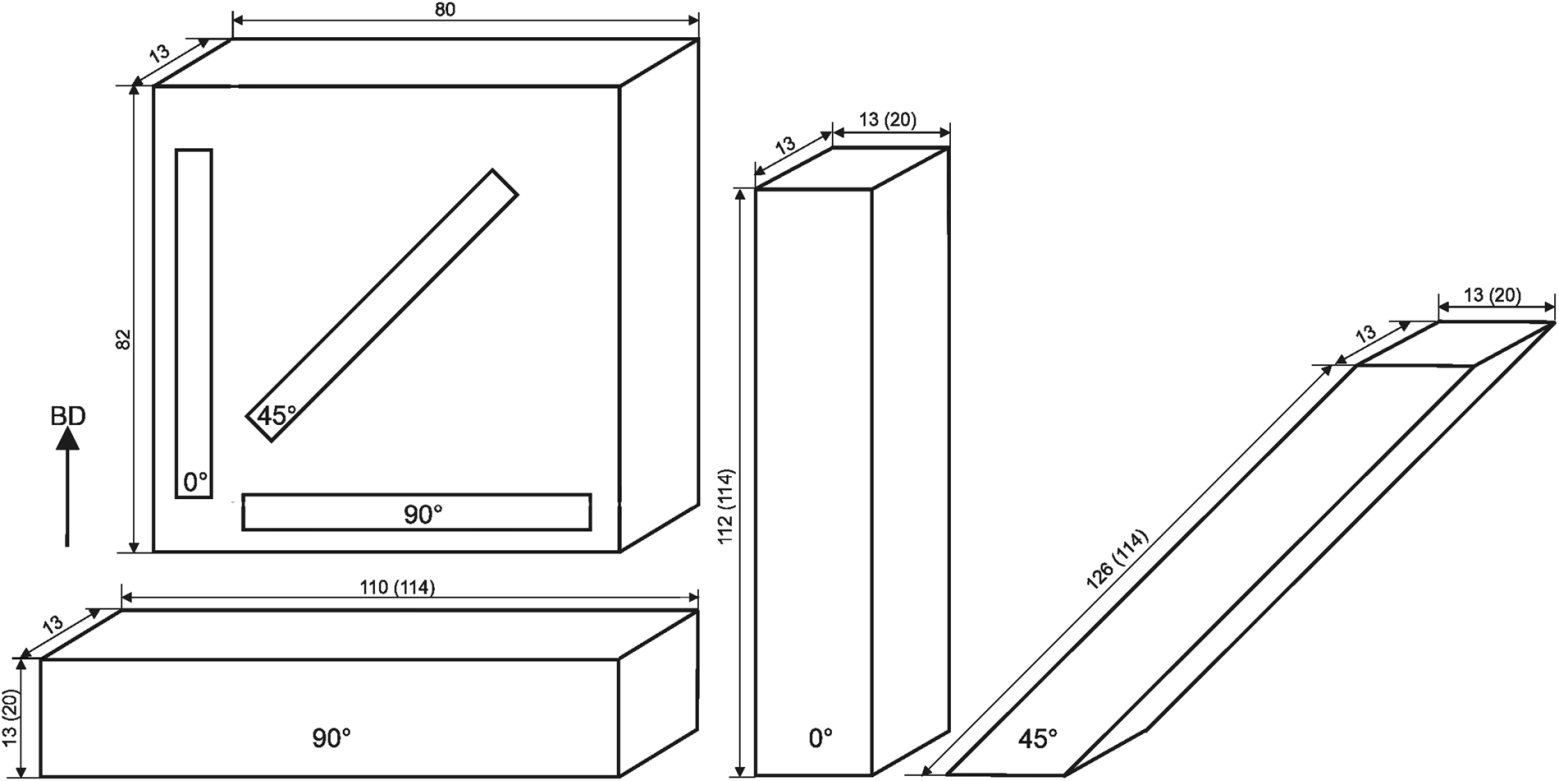
Schematic of AM building direction (BD) of towers and walls and the respective inclination of L-direction of the test pieces for elastic modulus measurements. For the towers, the L-direction is parallel to the longest direction of the blank.

Three different metallic alloys were investigated, each in an additively manufactured and a typical conventionally manufactured variant. In the AM variants, cylindrical towers (Ti-6Al-4 V) and rectangular towers and walls (IN718, AISI 316 L) were manufactured. AISI 316 L and IN718 blanks were manufactured via laser powder bed fusion (PBF-LB/M); Ti-6Al-4V blanks via directed energy deposition (DED-LB/M). The conventionally manufactured material variants were available as bars (Ti-6Al-4V, IN718) or plate (AISI 316 L). For the definition of the building/forming direction see Fig. 1. The specimen extraction direction (i. e. longitudinal (L-) direction) of the test pieces was in the case of the towers always parallel to the longest direction of the blank and in the case of the walls, in three directions, as indicated in Fig. 1.

#### Titanium alloy Ti-6Al-4V (material no. 3.7164)

All details on the manufacturing parameters, the chemical composition, and the microstructure of the laser powder-based directed energy deposition (DED-LB/M) processed material are given in^30,34^. The cylinders were manufactured on a TRUMPF TruLaser Cell 7020 machine (TRUMPF GmbH & Co. KG, Ditzingen, Germany). The cylindrical blanks had a diameter of 18 mm and a height of 123 mm and did not undergo any subsequent heat treatment.

Two variants of conventionally hot-formed material (cylindrical bars) were heat treated to obtain two different but commonly used microstructures (equiaxed, lamellar). Details are also summarized in^30,34^.

#### INCONEL® alloy 718 (material no. 2.4668; IN718)

Details on the manufacturing parameters of the laser powder bed fusion (PBF-LB/M) process are given in^31,35^. The specimens were manufactured on an SLM Solutions 280HL machine (SLM Solutions Group AG, Germany). Towers with their length direction in 0° (vertical, approx. dimensions 13 mm × 13 mm × 112 mm), 45° (diagonal, approx. dimensions 13 mm × 13 mm × 126 mm) or 90° (horizontal, approx. dimensions 13 mm × 13 mm × 110 mm) with respect to the build direction were used for modulus measurements. The actual chemical composition is given in Table 1.

**Table 1:** Chemical composition of PBF-LB/M variant of IN718 analyzed by optical emission spectrometry (OES).

Element	Ni	Cr	Fe	Nb	Mo	Ti	Al	Co	Mn	Si	Cu
Mass-%	54.3	17.82	18.36	4.38	3.46	0.92	0.48	0.07	0.09	0.05	0.03

Note: Difference to 100% - small amounts of other elements.

Conventionally processed material was investigated in the form of hot-rolled bar sections with a diameter of 16 mm. The chemical composition given in the supplier certificate is summarized in Table 2. The content of other elements such as C, B and P is less than 0.03%.

**Table 2:** Chemical composition of conventional IN718 according to supplier certificate (Enpar Sonderwerkstoffe GmbH, Gummersbach, Germany).

Element	Ni	Cr	Fe	Nb	Mo	Ti	Al	Co	Mn	Si	Cu
Mass-%	53.7	17.82	18.26	5.21	3.02	0.93	0.53	0.16	0.14	0.08	0.04

Note: Difference to 100% - small amounts of other elements.

Prior to the resonance measurements, material from both processing routes were heat treated (the PBF-LB/M material together with the base plate) with parameters as given in the Excel data file^35^. The objective was to achieve a relevant hardening condition.

#### Austenitic stainless steel AISI 316 L (material no. 1.4404; X2CrNiMo17-2-2)

Details on the manufacturing parameters, the chemical composition, and the microstructure of the laser powder bed fusion (PBF-LB/M) processed material and on the post-heat treatment are given in^20,29,36^. The specimens were manufactured on the same machine in the same laboratory at BAM as the IN718 (SLM Solutions 280HL machine, SLM Solutions Group AG, Germany). Two types of blanks were manufactured: walls (approx. dimensions: 13 mm × 80 mm × 82 mm) and towers (approx. dimensions: 13 mm × 20 mm × 114 mm). In addition to the PBF-LB/M process variants published in^20,29^, towers with a layer thickness of 30 µm were produced in three build directions. All variants are summarized in Table 3.

**Table 3:** Summary of powder layer thickness and specimen length direction (L) with respect build direction (BD) for PBF-LB/M/316 L.

Blank type	Layer thickness/µm	Inclination of specimen L-direction relative to BD
Tower	50	0°
Tower	30	0°
Tower	30	45°
Tower	30	90°
Wall	50	0°
Wall	50	45°
Wall	50	90°

A conventionally processed hot rolled sheet was also investigated for comparison. Details for the wrought material are also summarized in^36^.

Prior to the resonance measurements, material from both processing routes were heat treated (the PBF-LB/M material together with the base plate to relax residual stresses from PBF-LB/M process) with parameters as given in the Excel data file^36^.

#### Grain size determination

The grain sizes for Ti-6Al-4 V (DED-LB/M and conventional) and for the hot rolled 316 L sheet material given in^34,36^ were determined according to ASTM E112^37^ and DIN EN ISO 643^38^ by applying the line-cutting method. Grain size numbers were converted to grain diameters according to the standard. In all other cases, grain sizes were calculated from EBSD measurements. The threshold misorientation value for high angle grain boundaries was 15° and the minimum area for a single grain was approx. 1185 µm^2^.

### Resonance method

Young´s modulus and shear modulus were determined using the resonance method according to ASTM E 1875^27^. All measurements were performed on the same calibrated machine (Elastotron 2000, HTM Reetz, Berlin, Germany) in the same laboratory according to the mentioned standard and using the same analysis procedure. All specimens for the resonance measurements were machined in the same workshop with the specimen geometry and with the high requirements for plane parallelism and surface for these specimen geometries recommended in the standard. Both moduli were determined using the frequency of resonance peaks, dimensions, and density of the specimen. Hence, among the dimensions also the weight of each specimen was measured. For tests at high temperatures, the dimension is corrected using the coefficient of thermal expansion taken from several sources^39^ (https://www.valbruna.de/de/werkstoff/3.7164-3.7165.html, https://www.vdm-metals.com/fileadmin/user_upload/Downloads/Data_Sheets/Datenblatt_VDM_Alloy_718.pdf, https://www.preschstahl.de/files/wd/1.4404.pdf).

**Fig. 2:**
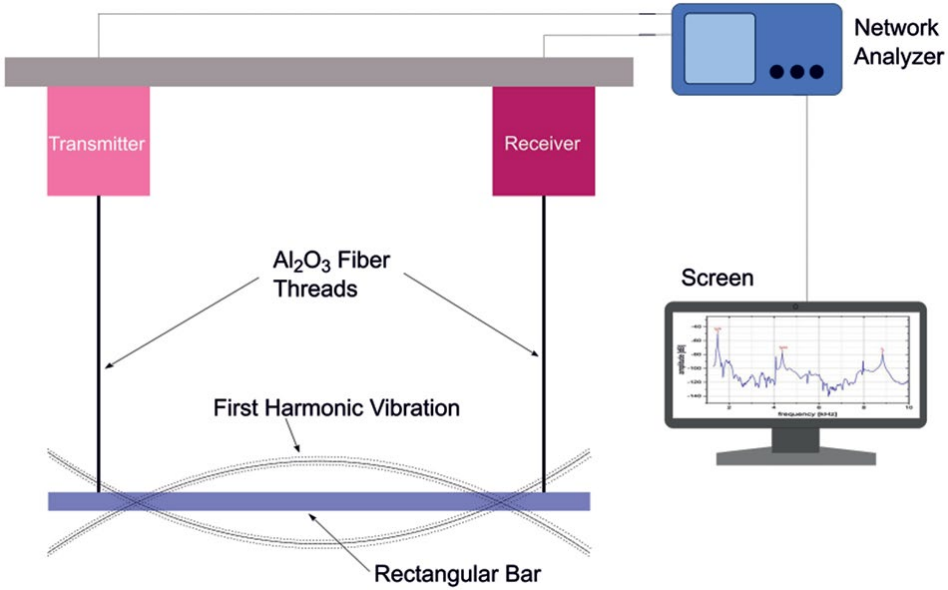
Schematic presentation of the experimental setup for dynamic modulus measurements by resonance technique.

To determine the resonance frequencies, a slim beam specimen was suspended between a piezoelectric transmitter and receiver using Al_2_O_3_ fiber threads. A sinusoidal signal from the transmitter vibrated the specimen and a detector (receiver) picked up the resulting oscillations. A schematic presentation of the experimental setup is shown in Fig. 2. For high temperature measurements the test setup included a carbon felt insulated furnace with graphite heating elements in a vacuum chamber.

**Fig. 3:**
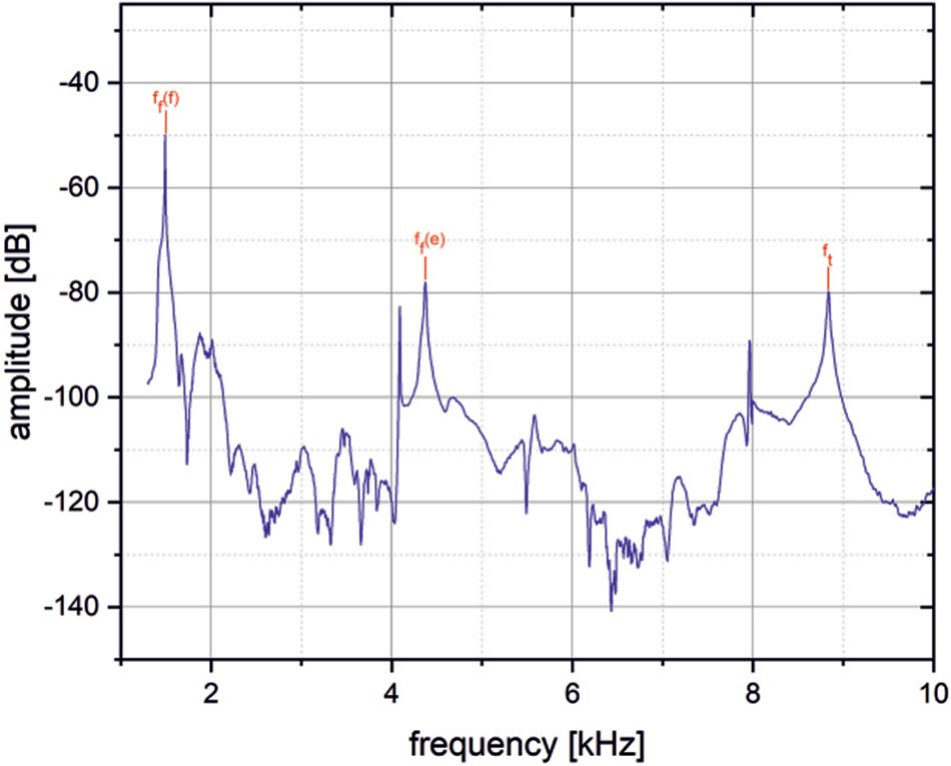
Example of resonance spectrum (PBF-LB/M/IN718 at room temperature).

The resonance spectrum was obtained by continuously varying the excitation frequency between 1 kHz and 70 kHz. The spectrum was recorded by a network analyzer. Analyzing the spectrum, the fundamental relevant vibrating frequency of the resonance bending peak, f_f_, and the torsional peak, f_t_, were determined. Figure 3 shows a part of a typical resonance spectrum with the characteristic vibration peaks.

The fundamental in flexure was recorded in both directions of the rectangular bar: edge-wise in the width direction, f_f_(e), and flat-wise in the thickness direction, f_f_(f). By determining the elastic modulus in both directions, it was possible to assess whether the material being tested behaves isotropically or anisotropically: different values in the two directions indicate anisotropy.

The Young´s modulus was calculated according to the standard and as given in Eq. (1) using the fundamental in flexure of a rectangular bar^27^:

1 $$E=0.9465\left(\frac{m{f}_{f}^{2}}{b}\right)\left(\frac{{L}^{3}}{{t}^{3}}\right){T}_{1}$$

with:

E Young´s modulus in units of Pa

L length of the bar in units of mm

b width of the bar in units of mm

t thickness of the bar in units of mm

m mass of the bar in units of g

f_f_ fundamental resonance frequency of the bar in flexure in units of Hz

T_1_ correction factor for fundamental flexural mode to account for finite thickness of the bar, Poisson´s ratio and so forth

2 $$\begin{array}{l}{T}_{1}=1+6.585(1+0.0752\mu +0.8109{\mu }^{2}){\left(\frac{t}{L}\right)}^{2}-0.868{\left(\frac{t}{L}\right)}^{4}\\ \,-\left[\frac{8.34(1+0.2023\mu +2.173{\mu }^{2}){(t/L)}^{4}}{1+6.338(1+0.1408\mu +1.536{\mu }^{2}){(t/L)}^{2}}\right]\end{array}$$

with:

µ Poisson´s ratio

The shear modulus was calculated according to the standard using the fundamental in torsion of a rectangular bar^27^:

3 $$G=\frac{4Lm{f}_{t}^{\,2}}{bt}[B/(1+A)]$$

with:

G shear modulus in units of Pa

f_t_ fundamental resonance frequency of the bar in torsion in units of Hz and

4 $$B=\left[\frac{\frac{b}{t}+\frac{t}{b}}{4\left(\frac{t}{b}\right)-2.52{\left(\frac{t}{b}\right)}^{2}+0.21{\left(\frac{t}{b}\right)}^{6}}\right]$$

A is an empirical correction factor. It depends on the width-to-thickness ratio, as follows:

5 $$A=\frac{0.5062-0.8776\left(\frac{b}{t}\right)+0.3504{\left(\frac{b}{t}\right)}^{2}-0.0078{\left(\frac{b}{t}\right)}^{3}}{12.03\left(\frac{b}{t}\right)+9.892{\left(\frac{b}{t}\right)}^{2}}$$

Dynamic techniques provide an advantage over static methods (like tensile tests) because of greater precision, ease of specimen preparation, and a wide variety of allowed specimen shapes and sizes^39,40^. For calculated measurement uncertainty of Young’s and shear modulus see section Technical Validation.

## Data Records

A separate Microsoft Excel *.xlsx file was created for each of the three alloys. The Excel data files are archived in the open-access data repository Zenodo^34–36^. Each Excel workbook is structured in the following spreadsheets: “nomenclature”, “process parameters”, “specimen overview”, “MU of measuring equipment” (MU = measurement uncertainty). Spreadsheets named after the specimen ID are given in the spreadsheet “specimen overview” and containing the data set for each of the measured specimens.

**Fig. 4:**
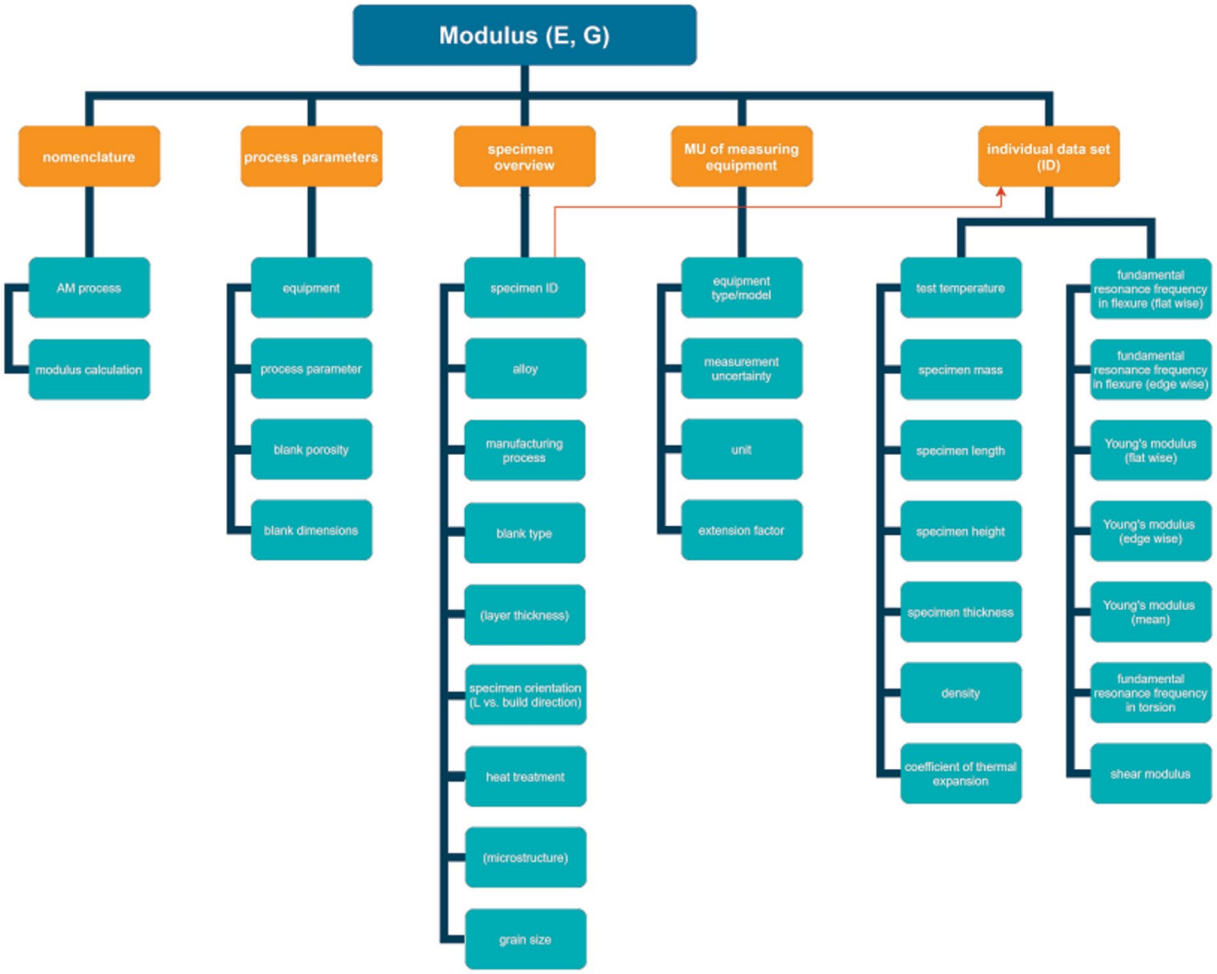
Overview of the data structure (in parentheses - if applicable). The red arrow indicates the link between the respective spreadsheets for each sample ID and the associated process and material information.

Each data set is composed of column data for the test temperature, specimen mass and dimensions, density at test temperature, coefficient of thermal expansion at test temperature, measured resonance frequencies of flexural and torsional mode as well as calculated Young’s modulus (incl. mean value of flat wise and edge wise measurement) and shear modulus. The data structure is illustrated in Fig. 4.

The temperature dependent Young’s and shear modulus can be plotted and interesting correlations (e.g. with build direction, microstructure) can be made with the data given in the data repository^34–36^.

It should be noted that no significant differences were found between flat-wise and edge-wise peaks for any of the three materials studied here, therefore macroscopic quasi-isotropy can be concluded.

## Technical Validation

All measuring equipment used (electronic balance, micrometer screw, caliper, network analyzer, thermocouples, data logger for calibration of temperature measuring chain) were calibrated, the expanded measurement uncertainties are given in the individual Excel data files for the three materials^34–36^, see spreadsheet “MU of measuring equipment”. In addition, a calibration procedure using an additional test piece was performed to optimize the temperature control and to check the temperature distribution along the longitudinal direction of the test piece. For this purpose, the temperature was increased stepwise by 600 K/hour until reaching the corresponding temperature level. The measurement at each temperature level started after a temperature-dependent soak time (between 10 and 25 minutes). The same heating rate and soak time was applied for the actual modulus measurements. The measurement uncertainty of the entire temperature measurement chain was calculated as max. 10 K.

The entire test setup is regularly checked with a reference specimen (cold work steel 115CrV3) for which the resonance spectrum is known.

The results for Young’s modulus from resonance measurement were compared (where possible) with measurements from high precision tensile tests. There was a very good agreement in the results. It should be noted that the elastic properties determined under adiabatic conditions exhibit slightly higher values compared to the elastic properties determined under isothermal conditions^40^.

The measurement uncertainty analysis of the dynamic Young’s modulus test based on a Code of Practice^41^. The calculations are consistent with ASTM E1875^27^. The calculated measurement uncertainty is max. 1% for room temperature and 3% for high temperature. The same approach is used to calculate the measurement uncertainty for the shear modulus, which results in max. 3% for room temperature and max. 5% at high temperature.

Finally, comparative measurements were performed using the pulse excitation method (according to ATME1876^42^) at room temperature, which showed very good agreement with the results obtained by the resonance method.

## Usage Notes

The data contained in the datasets may be used for various purposes. The basic usage may involve comparing modulus data for conventionally processed and additively manufactured materials. The data can be used to rate data produced in other laboratories and with the same or with different methods (considering similar material processing and heat treatment). The data also provide information on the dependence of the sampling direction relative to the build direction. The data can also be useful for mechanical testing in which the Young’s modulus is used to verify the correct test setup (e. g., low-cycle fatigue (LCF) or thermo-mechanical fatigue (TMF)). The modulus can also be useful for designing VHCF test pieces.

In addition, the data can be very useful for simulation purposes. Structural evaluation procedures often involve the simulation of critical structures in terms of structural integrity. This requires suitable input data, particularly of material properties such as elastic and shear modulus, as provided here. A solid database on material properties for additively manufactured materials under consideration for industrial applications is only now developing. The measurement of elastic properties under different process conditions thus represents an essential contribution to future simulation work.

## Data Availability

The software “Elastotron 2000” V.6 was used to analyze the resonance spectra. Details are given in section 2 in a publication by Kaindl *et al*.^43^. Because of the age of the instrument this software is not accessible, however any standard data analysis tool (Excel, Matlab, etc), or manual inspection, can be used to replicate its functionality.
